# Prediabetes remission after bariatric surgery: a 4-years follow-up study

**DOI:** 10.1186/s12902-024-01537-0

**Published:** 2024-01-11

**Authors:** Marta Borges-Canha, João Sérgio Neves, Maria Manuel Silva, Fernando Mendonça, Telma Moreno, Sara Ribeiro, Catarina Vale, Juliana Gonçalves, Helena Urbano Ferreira, Sara Gil-Santos, Raquel Calheiros, Inês Meira, João Menino, Vanessa Guerreiro, Jorge Pedro, Ana Sande, Selma B. Souto, Eduardo Lima da Costa, Davide Carvalho, Paula Freitas, CRIO group, John Rodrigues Preto, Hugo Miguel Santos Sousa, André Manuel Costa Pinho, Carla Cristina Oliveira Rodrigues Teixeira Galego, Maria Flora Ferreira Sampaio Carvalho Correia, Cidália Fátima Castro Gil, Diva Bizarro Figueiredo Melim, Eduardo Gil Ferreira Rodrigues Pinto, Marco António Costa Silva, Cristina Sarmento Pontes Martins, Luís Miguel Gonçalves Pereira, Inês Vasconcelos Sousa Magalhães, Isabel Maria Boavista Vieira Marques Brandão, Sertório Manuel Freitas Andrade, Patrícia Maria Lopes Nunes

**Affiliations:** 1 Serviço de Endocrinologia, Diabetes e Metabolismo do Centro Hospitalar Universitário de São João, Porto, Portugal; 2 Departamento de Cirurgia e Fisiologia, Faculdade de Medicina da Universidade do Porto, Porto, Portugal; 3 Serviço de Medicina Interna do Centro Hospitalar Universitário de São João, Porto, Portugal; 4 Serviço de Endocrinologia do Instituto Português de Oncologia do Porto, Porto, Portugal; 5 Serviço de Cirurgia Geral do Centro Hospitalar Universitário de São João, Porto, Portugal; 6 Instituto de Investigação e Inovação em Saúde (i3s), Faculdade de Medicina da Universidade do Porto, Porto, Portugal; 7 Centro Hospitalar Universitário de São João, Porto, Portugal

**Keywords:** Prediabetes, Glycaemic control, Bariatric surgery, Metabolic surgery

## Abstract

**Background:**

Bariatric surgery leads to weight loss and to cardiometabolic risk improvement. Although prediabetes remission after bariatric surgery is biologically plausible, data on this topic is scarce. We aimed to assess prediabetes remission rate and clinical predictors of remission in a 4 year follow up period.

**Methods:**

Observational longitudinal study including patients with obesity and prediabetes who had undergone bariatric surgery in our centre. Prediabetes was defined as having a baseline glycated haemoglobin (A1c) between 5.7% and 6.4% and absence of anti-diabetic drug treatment. We used logistic regression models to evaluate the association between the predictors and prediabetes remission rate.

**Results:**

A total of 669 patients were included, 84% being female. The population had a mean age of 45.4 ± 10.1 years-old, body mass index of 43.8 ± 5.7 kg/m^2^, and median A1c of 5.9 [5.8, 6.1]%. After bariatric surgery, prediabetes remission rate was 82%, 73%, 66%, and 58%, respectively in the 1st, 2nd, 3rd, and 4th years of follow-up. Gastric sleeve (GS) surgery was associated with higher prediabetes remission rate than Roux-en-Y gastric bypass surgery in the 3rd year of follow-up. Men had a higher remission rate than women, in the 1st and 3nd years of follow-up in the unadjusted analysis. Younger patients presented a higher remission rate comparing to older patients in the 3rd year of follow-up.

**Conclusion:**

We showed a high prediabetes remission rate after bariatric surgery. The remission rate decreases over the follow-up period, although most of the patients maintain the normoglycemia. Prediabetes remission seems to be more significant in patients who had undergone GS, in male and in younger patients.

**Supplementary Information:**

The online version contains supplementary material available at 10.1186/s12902-024-01537-0.

## Introduction

Prediabetes is a dysglycemic state that confers a high risk for overt type 2 diabetes (T2D), and is thought by most authors to be a precursor of T2D. People with prediabetes are at risk of the same microvascular and macrovascular complications (including cardiovascular risk) as patients with diabetes, although the risk is lower [1]. A lot of uncertainty regarding diagnostic criteria and treatment options and targets remain to be established [1].

The need for remission of prediabetes and for prevention of progression from prediabetes to T2D seems logical and has been largely studied through lifestyle modification and glucose-lowering drug approaches. The combined results show that diabetes incidence can be greatly reduced with these approaches [2, 3].

Little is known regarding the effect of bariatric surgery (BS) on the remission of prediabetes. BS is a highly effective treatment for obesity, which is associated with a significant cardiometabolic improvement, and has been proven to reduce cardiovascular risk both in patients with diabetes and prediabetes [4]. There is firm evidence regarding diabetes remission after BS and glucose metabolism improvement [5, 6]. Our group has previously studied predictors of diabetes remission after BS and concluded that patients age, preoperative glycated haemoglobin (A1c) and preoperative beta cell are useful for predicting diabetes remission [7]. In addition, Moriconi D. et al., showed that short T2D duration and good glycaemic control before BS were associated with long-lasting diabetes remission [8]. This is in accordance with data from Panunzi S. et al. [9]. However, data regarding prediabetes remission after BS is scarce, besides being biologically plausible. We believe that this is an important gap in knowledge, considering the possible benefit of bariatric surgery in prediabetes.

We hypothesized that BS is associated with a high and long-lasting prediabetes remission rate. Therefore, our primary aim was to assess pre-diabetes remission rate over a 4 year follow up period. Our secondary aim was to evaluate sex, age, and type of surgery as possible clinical predictors of diabetes remission rate in the same follow-up period.

## Methods

### Study design

This is a retrospective observational study evaluating all the patients with obesity and prediabetes who had BS and were followed in our tertiary centre between January 2010 and December 2021. Patients taking antidiabetic drugs were considered to have diabetes regardless of A1c.

### Study participants

**Fig. 1:**
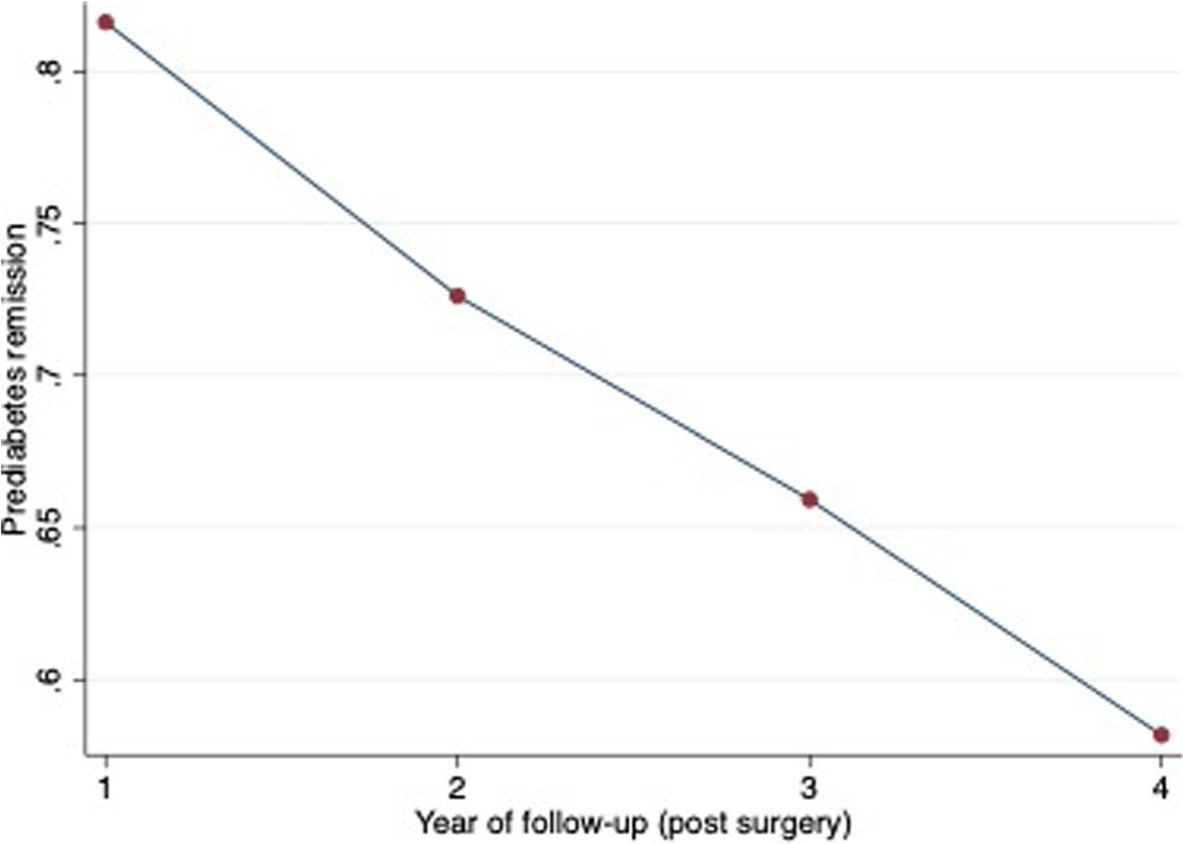
Flow chart of included population

Among the patients with prediabetes who had BS, we excluded those who had no A1c in the first year of follow-up or who had undergone gastric band surgery, while all the other patients were included in the analysis (Fig. 1). After applying the above exclusion criteria, a total of 669 patients were included in this analysis.

### Clinical definitions

Prediabetes was defined as A1c ≥ 5.7% and < 6.5% and no treatment with antidiabetic drugs [10]. Hypertension was defined as systolic blood pressure ≥ 140 mmHg, diastolic blood pressure ≥ 90 mmHg [11], or the use of anti-hypertensive drugs. Dyslipidemia was defined by the use of lipid-lowering agents, serum LDL cholesterol ≥ 160 mg/dL, serum HDL cholesterol < 40 mg/dL, or serum triglycerides ≥ 200 mg/dL [12]. Lost weight was calculated as a percentage of total weight loss (TWL) at each year after BS according to the formula: TWL=([Preoperative weight – Postoperative weight]/ Preoperative weight)×100) [13].

### Outcomes and statistical analysis

Continuous variables are described as mean ± standard deviation or median (25th -75th percentiles) and categorical variables as proportions (percentages). We performed unadjusted and adjusted logistic regression analysis considering prediabetes remission as the primary outcome. The adjusted model included sex, age, baseline A1c, type of surgery performed and lost weight. The model was built based on prior knowledge of risk factors/confounders and biological plausibility. We performed subgroup analysis to assess the associations of our predefined variables (sex, age, and type of surgery) with prediabetes remission. Statistical analyses were conducted using Stata software, version 14.1 (StataCorp).

## Results

### Baseline population characteristics

Table 1 shows the demographic and clinical characteristics of the included population (*n* = 669). The population had a mean age of 45.4 ± 10.1 years-old, body mass index of 43.8 ± 5.7 kg/m^2^, and median A1c of 5.9 [5.8, 6.1] %. The majority of patients (69%) underwent gastric bypass (GB) surgery. Supplementary Table 1 shows loss of follow-up per year (31%, 46% and 61% respectively for 2nd, 3^rd,^ and 4th years).

**Table 1:** Clinical and demographic characteristics of the population included (*n*=669)

Age, years	45.4 ± 10.1
Feminine sex, n (%)	561 (83.9)
Weight, kg	115.7 ± 19.2
Body Mass Index, kg/m^2^	43.8 ± 5.7
Waist circumference, cm	123.9 ± 13.2
Hip circumference, cm	131.7 ± 11.4
Dyslipidaemia, n (%)	299 (46.1)
Hypertension, n (%)	433 (66.6)
Glycated haemoglobin, %	5.9 ± [5.8, 6.1]
Type of surgery	
Gastric bypass, n (%)	460 (68.8)
Gastric sleeve, n (%)	209 (31.2)

Values are shown as mean ± standard deviation or as median [percentile 25 – percentile 75]

### Prediabetes remission after bariatric surgery

**Fig. 2:**
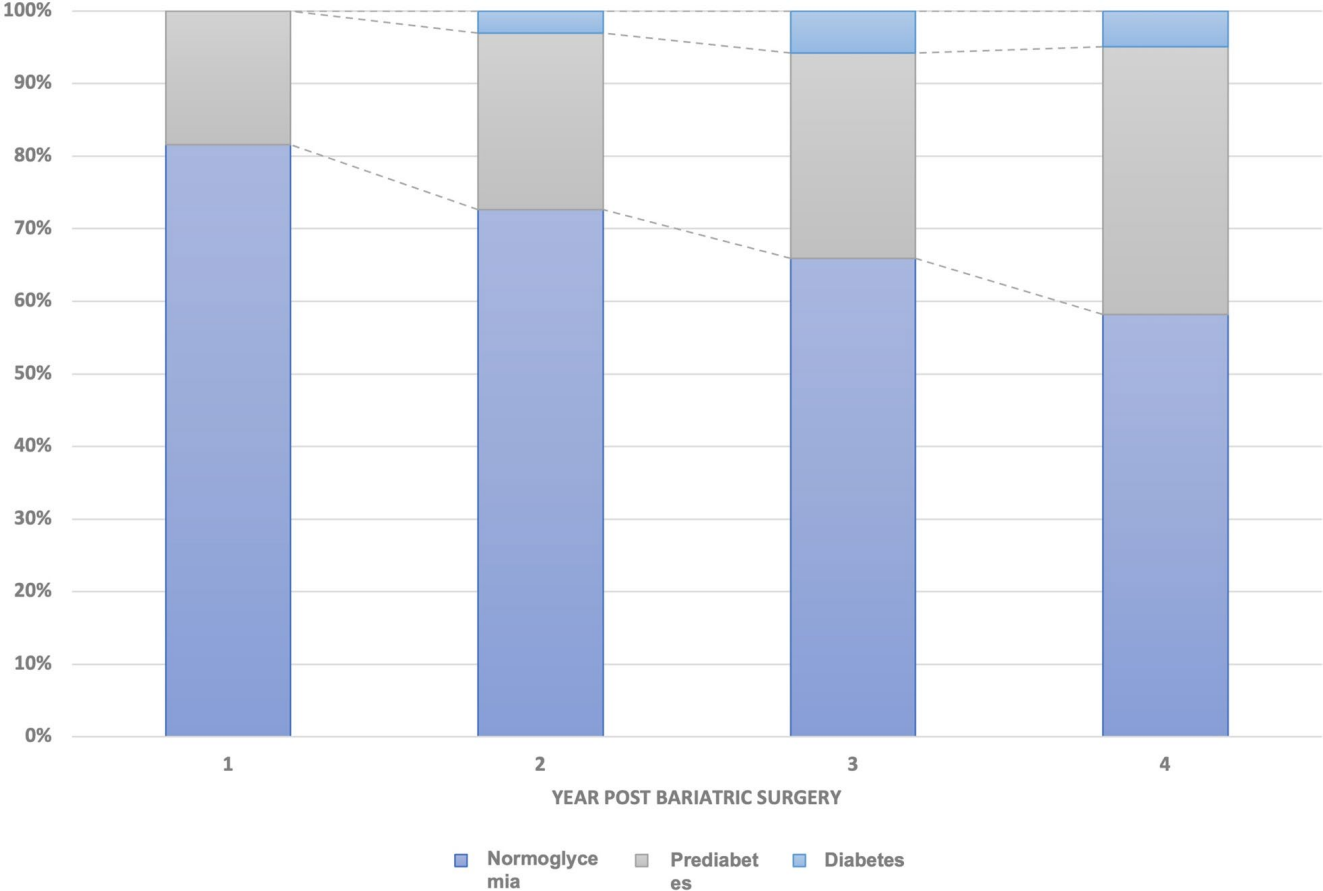
Prediabetes remission rate plot over time. Values are show as the percentage of prediabetes remission

Figure 2 depicts prediabetes remission rate over time. One year after BS there was a prediabetes remission rate of 82%, the same remission rate being 73%, 66% and 58% in the 2nd, 3rd and 4th years after BS, respectively.

**Fig. 3:**
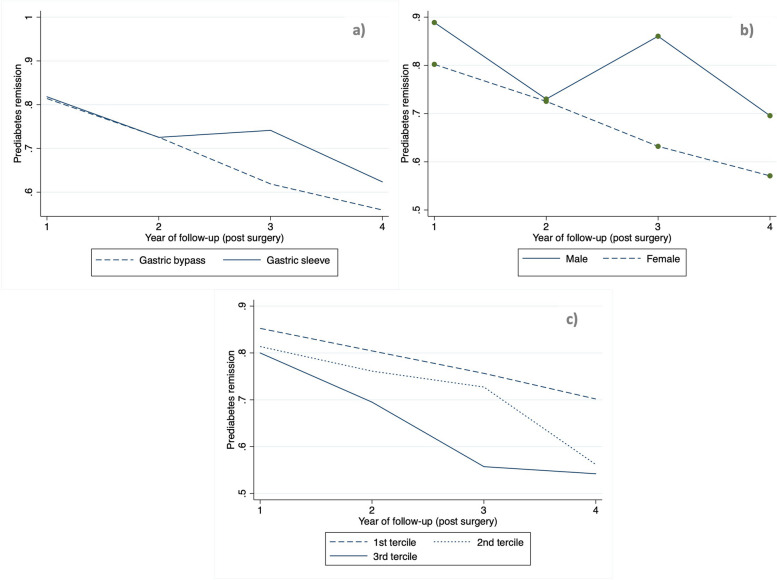
Evolution of patients with prediabetes to normoglycemia, prediabetes or diabetes along the follow-up

Figure 3 shows the evolution of patients with prediabetes at baseline to normoglycemia, prediabetes or diabetes along the follow-up. 24%, 28% and 37% are in the prediabetic range, and 3%, 6% and 5% in the diabetic range at the 2nd, 3rd, and 4th years post BS, respectively.

### Subgroup analysis

**Fig. 4:**
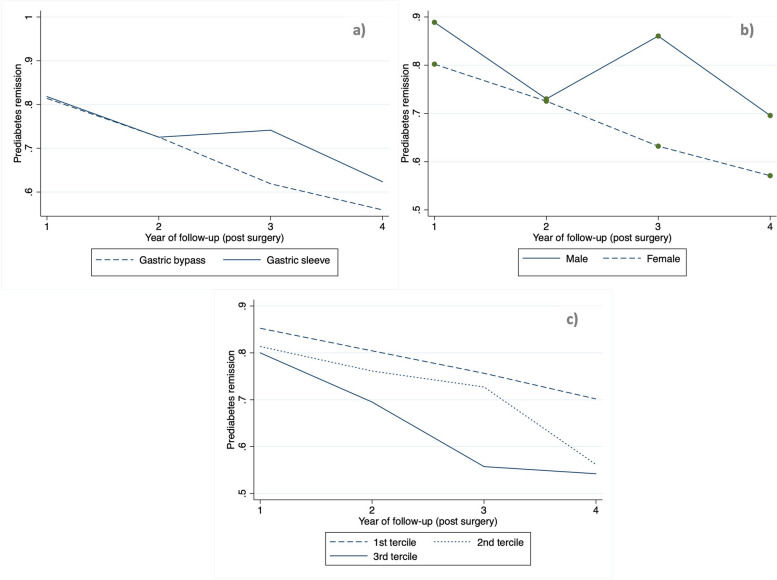
Prediabetes remission rates along the follow-up according to (**a**) type of surgery; (**b**) sex; (**c**) age tercile

Figure 4a; Table 2 show our subgroup analysis regarding remission of pre-diabetes according to the type of surgery. There are no differences between patients which underwent GB surgery and gastric sleeve (GS) surgery in the 1st and 2nd years post BS. Patients who had GS had a higher odds ratio for prediabetes remission in the 3rd year of follow-up both in adjusted and non-adjusted analysis. No differences were seen in the 4th year of follow-up.

**Table 2:** Remission of pre-diabetes according to the type of surgery (4 years of follow-up)

	**Unadjusted analysis**		**Adjusted analysis** ^a^	
	OR	95% CI	OR	95% CI
**Year 1**	1.02	0.67 – 1.56	0.78	0.45 – 1.34
**Year 2**	0.99	0.64 – 1.55	1.54	0.86 – 2.75
**Year 3**	1.75	1.08 – 2.86	2.02	1.13 – 3.61
**Year 4**	1.29	0.76 – 2.19	1.60	0.85 – 3.00

^a^Adjusted models: sex, age, weight lost, glycated haemoglobin

*OR* Odds ratio, *CI* Confidence Interval

Figure 4b; Table 3 refer to remission of pre-diabetes according to sex. Female patients present a lower odds ratio for prediabetes remission in the 1st and 3rd years of follow-up, only in the non-adjusted analysis.

**Table 3:** Remission of pre-diabetes according to sex (4 years of follow-up)

	**Unadjusted analysis**		**Adjusted analysis** ^a^	
	OR	95% CI	OR	95% CI
**Year 1**	0.51	0.26 – 0.95	0.46	0.19 – 1.08
**Year 2**	0.98	0.54 – 1.77	1.05	0.48 – 1.01
**Year 3**	0.28	0.11 – 0.68	0.49	0.19 – 1.30
**Year 4**	0.58	0.23 – 1.47	0.49	0.17 – 1.39

^a^Adjusted models: age, type of surgery, weight lost, glycated haemoglobin.

*OR* Odds ratio, *CI* Confidence Interval

Figure 4c; Table 4 concern the remission of pre-diabetes according to age terciles (1st tercile, 19–38 years-old; 2nd tercile 39–48 years-old; 3rd tercile 49–67 years-old). Older patients (3rd tercile compared to 1st tercile) have a lower odds ratio for pre-diabetes remission throughout follow-up in the non-adjusted analysis from 3rd year of follow-up onwards; this is significant only for the 3rd year in the adjusted analysis.

**Table 4:** Remission of pre-diabetes according to age terciles (4 years of follow-up; 1^st^ tercile as comparer)

	**Unadjusted analysis**				**Adjusted analysis** ^a^			
	2^nd^ tercile		3^rd^ tercile		2^nd^ tercile		3^rd^ tercile	
	OR	95% CI	OR	95% CI	OR	95% CI	OR	95% CI
**Year 1**	0.76	0.40 – 1.43	0.69	0.37 – 1.29	0.87	0.45 – 1.70	0.90	0.47 – 1.73
**Year 2**	0.78	0.40 – 1.49	0.55	0.30 – 1.04	0.89	0.45 – 1.76	0.72	0.36 – 1.40
**Year 3**	0.86	0.44 – 1.67	0.41	0.22 – 0.75	0.95	0.48 – 1.90	0.41	0.21 – 0.80
**Year 4**	0.54	0.27 – 1.10	0.50	0.35 – 0.996	0.75	0.35 – 1.61	0.62	0.29 – 1.31

1^st^ tercile, 19-38 years-old; 2^nd^ tercile 39-48 years-old; 3^rd^ tercile 49-67 years-old

^a^Adjusted models: sex, type of surgery, weight lost, glycated haemoglobin

*OR* Odds ratio, *CI* Confidence Interval

## Discussion and conclusions

This is a longitudinal study including patients with obesity and prediabetes who underwent to bariatric surgery, with the aim of assessing the pre-diabetes remission rate and the clinical predictors of remission over a 4 year follow up period. Our results show a high prediabetes remission rate after bariatric surgery, which is more significant in patients who have undergone GS surgery, in male and in younger patients. These results help to fill the gap in knowledge regarding the effect of bariatric surgery on prediabetes.

We report an 82% remission rate of prediabetes in the first-year post BS, and remission rates of 73%, 66% and 58%, respectively, in the 2nd, 3rd and 4th years post BS. In addition, our results show that 3%, 6% and 5% of patients developed diabetes, respectively in the 2nd, 3rd, and 4th years post BS. Although specific data on prediabetes remission after BS is lacking, there is evidence supporting that BS leads to significant long-term improvement in fasting plasma glucose levels among an ethnically diverse population [5]. Concerning diabetes conversion in patients with prediabetes, Dicker et al. aimed to assess conversion rate of prediabetes to diabetes in a 5 year-follow up after BS and reported a 10.1% conversion rate [14]. The difference between this conversion rate and ours might be due to several reasons, such as the different populations studied and the difference in follow-up period.

With regard to the subgroup analyses, our results show that patients who underwent GS surgery are more prone to prediabetes remission, comparing to patients who underwent GB in the 3rd year of follow up. We believe that the lack of significant results at 4th year is due to the the missing data. Although there is a clear gap in knowledge in this regard, there are data comparing the two procedures considering overt T2D remission. However, results are quite controversial. Some authors argue that both surgical procedures are associated with a similar diabetes remission rate [15, 16], while others show that GB is a better option comparing to GS [17–19]. GB has long been regarded as the gold-standard procedure in surgical treatment of obesity and its related comorbidities. However, GS is being more frequently performed due to its easier and potentially safer technique [20]. There are few studies comparing the two regarding the outcomes and our study contributes to the missing information. Peteril et al. report no significant difference between the two procedures concerning excess BMI loss at 5 years of follow-up after BS [20].

Additionally, we report that male patients have a higher odds ratio for prediabetes remission in the 3rd year post BS only in the non-adjusted analysis (no differences were noticed in the first two years post BS nor at 4th year). According to our results, Mousapour et al. did not report any sex differences in overt diabetes remission in males and females 1 year post BS; however, these authors did not present the longer-term follow-up period analysis [13]. Concerning sex disparities, women undergo BS more often than men, for reasons that are not entirely understood. It is likely that greater body image frustration plays an important role, and limited data on outcomes and complications may also be barriers to BS in man [21–23]. For instance, there are also controversial results on the disparities between the sexes with regard to weight loss reporting advantage both for females and for males in different studies [24–26]. We believe that our results are helpful in proving the benefit of BS in remission of prediabetes in men.

We hint that younger patients have a higher prediabetes remission rate than older patients, regardless of sex, type of surgery, lost weight, baseline A1c. These results are in accordance with the existing literature which is consistent with the fact that younger individuals tend to benefit more from BS. Contreras et al. showed that weight loss decreases with age [27]. Major P et al. also report lower weight loss for patients over 50 years old; however, it is noteworthy that BS is effective and safe in these patients as well [28]. It is hypothesized that the lower benefit of older individuals may result from a decrease in metabolic rate, functional status, and physical activity, which are inherent to the ageing process [28].

There are inherent limitations to this study which must be acknowledged. Firstly, this is a longitudinal retrospective study and, as such, more prone to specific bias. For occasion, we do not have fasting glucose data and, so prediabetes was defined solely based on A1c. Also, we have a considerable amount of missing data due to the loss to follow-up from the 3rd year after BS onwards. This may lead to selection bias affecting our results and may explain why some results do not reach statistical significance. Furthermore, there are possible confounders that we may have not accounted with, although we believe that our model of adjustment includes the most relevant ones. However, the importance of our results and the number of individuals included from this often-overlooked population fairly overcome these limitations and opens a door in this area of knowledge. Our data can help physicians counsel patients with prediabetes regarding the high probability of remission after BS, as well as predicting the probability of remission according to the type of surgery, age and sex.

Concluding, our results show a high prediabetes remission rate after BS, which decreases over the follow-up period, although most patients maintain the normoglycemia. Prediabetes remission seems to be more significant in patients who had undergone GS, in male and in younger patients. These results help to fill the knowledge gap on the effect of bariatric surgery in glucose metabolic disturbances.

### Supplementary Information

**Additional file 1: Supplementary Table 1. **Loss of follow-up per year.

## Data Availability

The dataset for this study is available from the corresponding author on reasonable request and ethical approval.

## Abbreviations

A1c: Glycated haemoglobin
BS: Bariatric surgery
GB: Gastric bypass
GS: Gastric sleeve
T2D: Type 2 diabetes
TWL: Total weight loss
