# Supplementary material for: Prediabetes remission after bariatric surgery: a 4-years follow-up study
Source: BMC Endocr Disord. 2024 Jan 11;24:7. doi: 10.1186/s12902-024-01537-0 (PMC10782579; doi:10.1186/s12902-024-01537-0)
Supplement: Supplementary file 1 — Additional file 1: Supplementary Table 1. Loss of follow-up per year. [file 12902_2024_1537_MOESM1_ESM.docx]

| **Supplementary Table 1.** Loss of follow-up per year | | | | |
| --- | --- | --- | --- | --- |
|  | Missing | Total | Percent missing |  |
| **Year 1** | 0 | 669 | 0 |  |
| **Year 2** | 209 | 669 | 31.24 |  |
| **Year 3** | 308 | 669 | 46.04 |  |
| **Year 4** | 406 | 669 | 60.69 |  |
